# Miscellany

**Published:** 1899-11

**Authors:** 


### MISCELLANY.

The Antikamnia Chemical Company, of St. Louis, has lately sent out to the profession a morocco pocket folder, which is a most convenient kind of pocket-book, intended especially for bank notes. The folder contains specimens of two new tablets that have just been formulated and put upon the market by this enterprising house. They are called the "antikamnia laxative tablets," and the "antikamnia and quinine laxative tablets." The formulæ of these tablets are as follows :

*Antikamnia Laxative Tablets.*

Each tablet contains :

| | |
|---|---|
| Antikamnia | gr. 4 3-4 |
| Cascarin | gr. 1-8 |
| Aloin | gr. 1-32 |
| Ext. belladonna | gr. 1-32 |
| Podophyllin | gr. 1-32 |

*Antikamnia and Quinine Laxative Tablets.*

Each tablet contains :

| | |
|---|---|
| Antikamnia | gr. 3 |
| Quinine bisulph | gr. 1 3-4 |
| Cascarin | gr. 1-8 |
| Aloin | gr. 1-32 |
| Ext. belladonna | gr. 1-32 |
| Podophyllin | gr. 1-32 |

The indications for their administration will readily occur to every intelligent physician and their convenience, as well as elegance, commend them as an addition of value to the therapeutic armamentarium of the profession.

